# A New Process of Direct Zinc Oxide Production by Carbothermal Reduction of Zinc Ash

**DOI:** 10.3390/ma15155246

**Published:** 2022-07-29

**Authors:** Jianjun Gao, Hong Wang, Jie Wang, Yingyi Zhang, Feng Wang, Shuang Yang, Shinan Li

**Affiliations:** 1 Research and Development Department, Gangyan Shenghua Engineering & Research Co., Ltd., Beijing 100081, China; gaojianjun2085@163.com (J.G.); flywise@126.com (F.W.); 2 State Key Laboratory for Advanced Iron and Steel Processes and Products, Central Iron and Steel Research Institute, Beijing 100081, China; 3 School of Metallurgical Engineering, Anhui University of Technology, Maanshan 243002, China; wanghong0531@126.com (H.W.); mrwang0101@ahut.edu.cn (J.W.); yangshuang20031003@163.com (S.Y.); lishinan031103@163.com (S.L.); 4 Beijing Metallurgical Equipment Research Design Institute Co., Ltd., Beijing 100029, China

**Keywords:** zinc ash, carbon-bearing pellet, roasting dechlorination, direct reduction, zinc oxide

## Abstract

Zinc ash is a by-product of the hot-dip galvanizing process and the electrolytic zinc process, which is classified as a hazardous waste consisting predominately of zinc oxide that could be recovered as the useful main resource for ZnO preparation. In this work, in order to reduce the energy consumption of the direct reduction process and improve the resource-recovery rate. A new technology for zinc oxide production, by a carbothermal reduction of zinc ash, is proposed. This process includes two steps: high-temperature roasting of zinc ash for dechlorination and a carbothermal reduction of dechlorination ash. Zn in zinc ash is mainly presented in the form of zinc oxide (ZnO), basic zinc chloride (Zn_5_(OH)_8_Cl_2_H_2_O), and metallic zinc (Zn). Basic zinc chloride can be roasted and decomposed to reduce the chlorine content in zinc ash. The results of a chloride ion removal test show that the optimal roasting temperature is 1000 °C, with a holding time of 60 min. Under the modified conditions, the chloride content in the roasted zinc ash is reduced to 0.021 wt.%, and the dechlorination rate is more than 99.5%, which can meet the requirements of zinc oxide production. The best process conditions for zinc oxide production by carbothermic reduction are as follows: reduction temperature of 1250 °C, reduction time of 60 min, and reduction agent addition of 22 wt.%. Under the best reduction process, the purity of zinc oxide product is 99.5%, and the recovery of zinc is more than 99.25%. Needle-like zinc oxide obtained by carbothermic reduction has high purity and can replace zinc oxide produced by an indirect process.

## 1. Introduction

Zinc oxide as one of important basic raw chemicals, which is widely used in rubber, paints, ceramics, and other chemical industries [1,2,3,4,5,6,7,8,9]. In China, the consumption of zinc oxide is 0.3~0.5 million tons per year, accounting for 5%~10% of the total amount of zinc [10,11]. The preparation of ZnO can be divided into the direct process, indirect process, and wet-chemical method (active zinc oxide). As raw material in the indirect process, metal zinc ingot or zinc slag is converted into zinc vapor in a graphite crucible at high temperature and then oxidized by pumped air. The obtained zinc oxide particles are transported via a cooling duct and collected, with a purity of 99.7%. The direct process involves the reduction of zinc ash or zinc oxide ore (raw material) by heating with coal in an open-hearth furnace, followed by the oxidation of zinc vapor and the bag collection of ZnO with a purity of 95%~99% [12]. For the wet-chemical method (active zinc oxide), secondary zinc oxide or zinc containing dust ash is used as a raw material to be leached out by ammonium bicarbonate and ammonia, to dissolve zinc oxide to form zinc-ammonium complex; then, the solution is purified and roasted to obtain the active zinc oxide with a purity of about 95%. Since hydrometallurgy will produce a large amount of wastewater containing heavy metal ions, pyrometallurgy is widely used to recover zinc oxide in China.

At present, an open-hearth furnace (OHF, also called a W-Zinc-Oxygen-Furnace) is used to recover zinc oxide from electric furnace ash. This smelting equipment has the following advantages: simple equipment, low investment, short construction period, and convenient operation. However, the traditional process has a long smelting cycle, intermittent operation, high fuel consumption, low energy efficiency, and serious pollution, which seriously restrict its popularization and application. Therefore, it is urgent to develop a new zinc oxide recovery technique [13,14,15,16,17]. In order to solve the above technical problems, the Central Iron and Steel Research Institute proposed a new technology, of direct zinc oxide production by carbothermal reduction. This technology has the following characteristics: energy saving, environmental protection, high efficiency, and automation. Compared with the conventional OHF, its specific coal consumption per ton of zinc oxide products could be reduced by more than 50%, the zinc recovery rate increases by over 5%, and the ZnO purity is up to 99%. In this work, the effects of direct reduction temperature, time, and reductant content on reduction rate of zinc oxide were analyzed and discussed. In addition, the phase composition and microstructure of zinc oxide products recovered by direct reduction were also investigated.

## 2. Experimental

### 2.1. Materials

**Figure 1:**
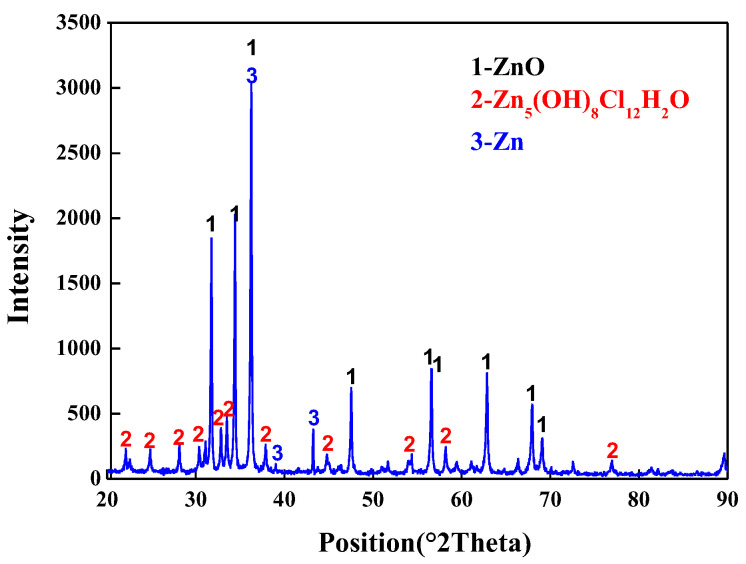
XRD pattern of zinc ash.

**Figure 2:**
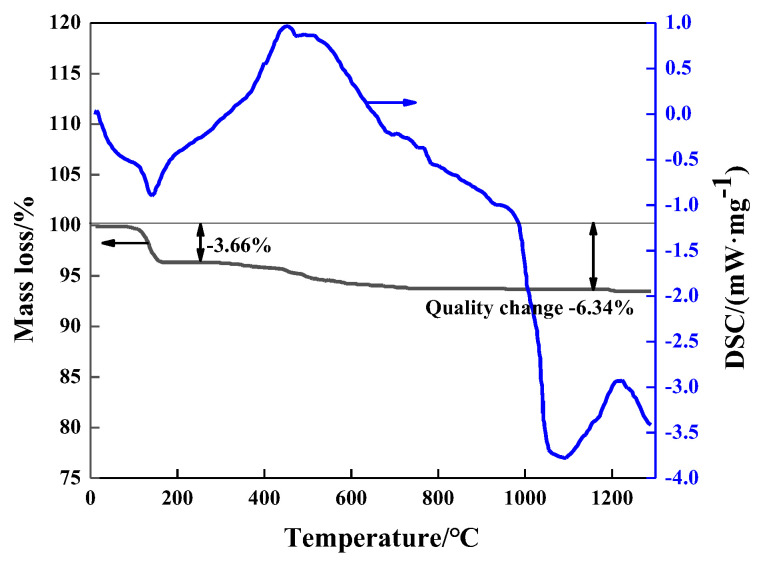
TG-DSC analysis of zinc ash.

The experimental raw material is zinc dust produced by hot-dip galvanizing, and the chemical composition of zinc ash is shown in Table 1. It can be seen that the contents of ZnO, Cl, and SiO_2_ are 87.91 wt.%, 4.44 wt.%, and 1.27 wt.%, respectively, while the contents of other impurities are low. It is worth noting that volatile Cl elements should be removed, otherwise, Cl will enter ZnO products in the form of ZnCl_2_, affecting the product quality. Figure 1 shows the XRD pattern of zinc ash. It can be seen that zinc ash is mainly composed of Zn (Ref. code 01-087-0713) and ZnO (Ref. Code 01-089-0511), with a small amount of Zn_5_(OH)_8_Cl_2_H_2_O (Ref. Code 01-077-2311). The Zn_5_(OH)_8_Cl_2_H_2_O is formed by the combination of Cl with zinc oxide and trace metal zinc. TG-DSC analysis of zinc ash (Figure 2) indicates that during the temperature-rising period, considerable mass loss of zinc ash occurs twice, with the first at about 130 °C and the second at about 500 °C. The total mass loss reaches 6.34%, as the temperature rises up to 1300 °C. Anthracite is used as the reducing agent, with a size of under 74 μm. As listed in Table 2, the fixed carton of coal powder is 78.73%, and the ash and volatile content are 10.16% and 8.55%, respectively. In addition, small amounts of S (0.28%) and P (0.023%) are also detected in anthracite.

### 2.2. Thermodynamic Basis for Reduction

Zn is mainly presented in the forms of ZnO, Zn_5_(OH)_8_Cl_2_H_2_O, and metallic Zn. Due to a certain Zn_5_(OH)_8_Cl_2_H_2_O in zinc ash, Cl needs to be removed by high-temperature roasting with the following chemical reactions.

Zn_5_(OH)_8_Cl_2_H_2_O = 4Zn(OH)_2_ + ZnCl_2_ + H_2_O_(g)_ (1)

Zn(OH)_2_ = ZnO + H_2_O_(g)_ (2)

ZnCl_2_ = ZnCl_2(g)_ (3)

As indicated in TG-DSC analysis (Figure 2), the first mass loss occurs in Reactions (1) and (2), where Zn_5_(OH)_8_Cl_2_H_2_O is decomposed to Zn(OH)_2_, ZnCl_2_, and H_2_O, and, at the same time, Zn(OH)_2_ further breaks into ZnO and H_2_O, with 3.66% mass loss at 130 °C. The second mass loss happens in Reaction (3) because of the volatilization of ZnCl_2_, with a boiling point at 732 °C in standard conditions. During the actual operation, ZnCl_2_ starts to volatilize at a temperature less than the boiling point, due to its lower vapor partial pressure than that in the normal standard conditions, and, with the rising temperature, ZnCl_2_ in the zinc ash vapors into the flue gas to reduce the Cl content in the zinc ash. After dechlorination, Zn in zinc ash is presented mainly in the form of ZnO. Zinc ash is mixed with coal powder to prepare the carbon-containing pellets with reactions at high temperatures, as follows:

ZnO + C = Zn_(g)_ + CO_(g)_       Δ*G^θ^* = 363,841 − 297*T* (4)

Under standard conditions, the reduction between ZnO and carbon could be fulfilled at above 1225 K, so the higher temperature is, the quicker the reaction. To speed up the reaction and improve Zn recovery, direct reduction temperature could be increased. The reduced zinc vapor goes, along with flue gas, into oxidation room and is oxidized into ZnO by the blasted air. Since the oxidization of zinc vapor is exothermic reaction, the temperature of flue gas in the oxidation room is intensely high, requiring special cooling device to decrease the flue temperature so that bag collector could be adopted to gather ZnO products.

### 2.3. Experimental Method

**Figure 3:**
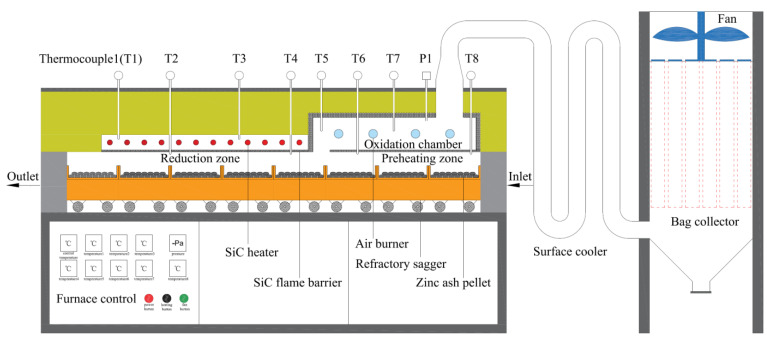
Experimental installation diagram of carbon thermal reduction of zinc ash.

As shown in Figure 3, the zinc oxide was produced by carbothermic reduction in an experimental silicon carbide heating furnace. The preparation process of zinc oxide includes four steps: zinc ash direct reduction, zinc vapor oxidization, surface cooling of ZnO flue gas, and bag collecting of ZnO products. To recover the immense amount of oxidization heat caused by zinc vapor and reduced CO oxidization and cut down the reduction energy consumption, the oxidation room is designed at the top of preheating area of zinc ash pellets to utilize the oxidation heat for preheating the pellets through radiation. The dechlorinated zinc ash is evenly mixed up with reduction agent at the presumed ratio, ground to −74 μm. The industrial syrup (5% of the mixture weight) is used as a binder, and then pressed into oval pellets with a size of 35 mm × 20 mm × 15 mm by briquetting machine. After being dried in drying box for one hour under 150 °C, the pellets are charged into a refractory sagger trolley with the stacking height of 50 mm and slowly pushed into the reduction furnace from the charging end. The pellets, after entering the reduction area through preheating area, are heated for a certain period at the set temperature before discharging. Under negative pressure, the reduced zinc vapor goes into oxidation room for ZnO oxidization by the blasted air. The resulting zinc oxide flue gas is cooled by surface cooler under 200 °C, and then ZnO products are gathered by bag collector. By weighing the reduced residue and ZnO product, analyzing Zn content to calculate the Zn-reduction rate, and by investigating the effects of reduction temperature, reduction time, reduction dosing, and other factors in zinc recovery, the optimal process parameters for the direct reduction of zinc ash to recover high-purity zinc oxide were finally determined.

## 3. Results

### 3.1. Zinc Ash Dechlorination Experiment

**Figure 4:**
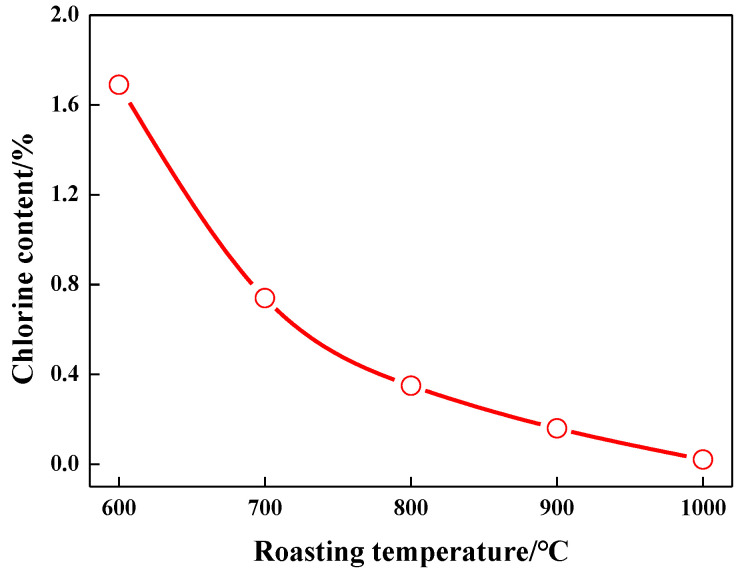
Effect of roasting temperature on chlorine content of zinc ash.

**Figure 5:**
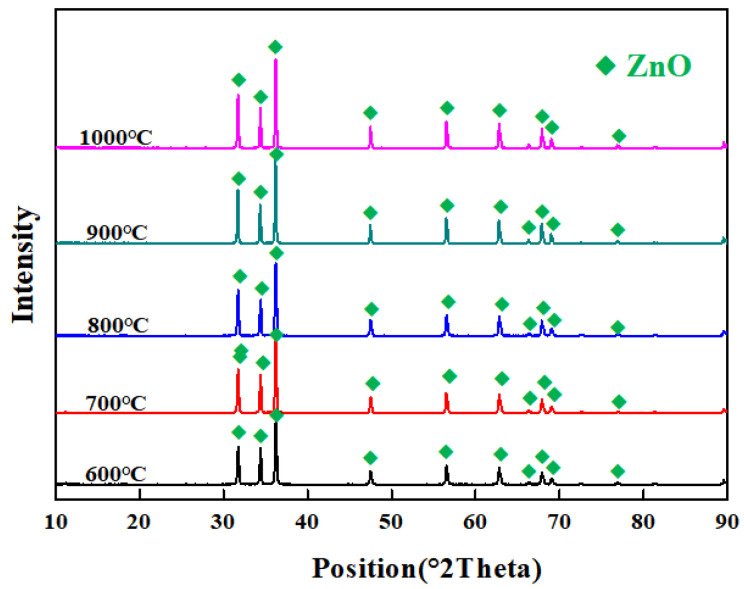
XRD pattern of zinc ash after dechlorination at different roasting temperatures.

The aim of zinc ash dechlorination is to remove Cl to obtain low-Cl zinc ash for the production of high-grade ZnO. According to the results from TG-DSC analysis of zinc ash, a roasting chloride-removal duration of 60 min is set. The influences on zinc ash dechlorination by different roasting temperatures (600 °C, 700 °C, 800 °C, 900 °C, and 1000 °C) are investigated, as shown in Figure 4. In addition, the corresponding XRD results are presented in Figure 5. It can be seen that no other impurities are observed in the zinc oxide products obtained at different temperatures, which is due to the low content of chloride.

As we know, Cl content in zinc ash is incredibly reduced, thanks to the decomposition of Zn_5_(OH)_8_Cl_2_H_2_O in zinc ash at high temperature and the breakdown of Zn(OH)_2_ to ZnO [18,19,20,21,22]. It can be seen from Figure 4 that the Cl content in zinc ash is 1.69% after being dechlorinated at 600 °C, which is mainly attributed to the low volatile temperature. When the temperature goes up to 700 °C, the Cl content is 0.74%, and the partial ZnCl_2_ is not fully volatilized, since its boiling point is at 732 °C. When the temperature rises to 800 °C, the Cl content in zinc ash is lowered to 0.35%, and the dechlorination rate is 90%. When the temperature is increased to 900 °C, the Cl content decreases to 0.16 wt.%. Although the Cl content has decreased significantly, the ZnO products are still not qualified. When the temperature grows to 1000 °C, the Cl content in zinc ash is 0.021 wt.% with a dechlorination rate of 99.5%, which completely meets ZnO production, as shown in Table 3. The removed chlorine exists in the form of ZnCl_2_ in the flue gas. ZnCl_2_ can be dissolved into the aqueous solution, by washing the flue gas to further prepare ZnCl_2_ products. As listed in the chemical analysis of zinc ash, after chloride removal, ZnO content ascends to 95.05%. The optimal conditions for dechlorination are determined as a roasting temperature of 1000 °C and a roasting time of 60 min.

### 3.2. Effect of Temperature on Reduction Rate of Zinc Oxide

**Figure 6:**
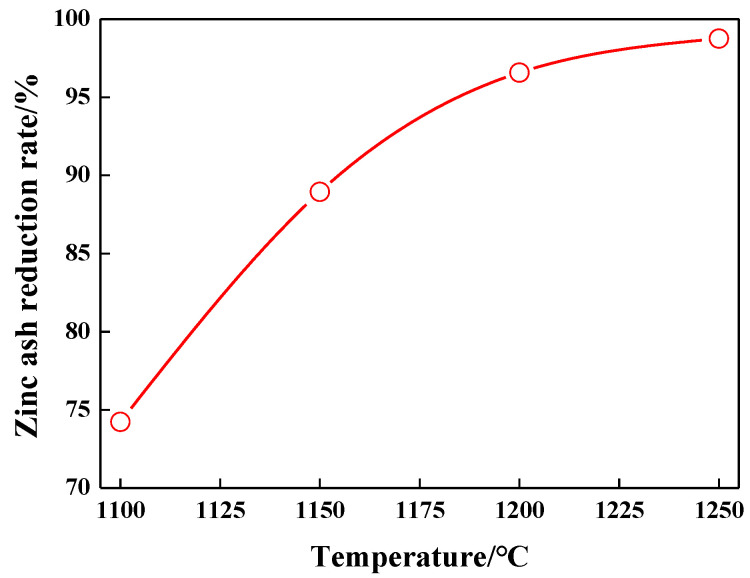
Effect of temperature on reduction rate of zinc oxide.

Reduction temperature affects not only the reduction rate of Zn in carbon-containing pellets but also the heating mode and energy consumption for smelting. The effects on the ZnO-reduction rate in carbon-containing zinc ash pellets are studied at different reduction temperatures (1100 °C, 1150 °C, 1200 °C, and 1250 °C) under the conditions of a 22% reduction agent dosage and a 40 min reduction duration, with the test results diagrammed in Figure 6.

As shown in Figure 6, reduction in carbon-containing zinc ash pellets increases gradually with the rising reduction temperature, so the higher the reduction temperature is, the greater the Zn-reduction rate. When the reduction temperature is 1100 °C, the reduction rate of Zn is only 74.23%. A layer of white zinc oxide powder was observed on the surface of the reduced pellet, and the low reduction rate and zinc oxide evaporation rate were mainly attributed to the low reduction temperature and vapor pressure. When the reduction temperature increases up to 1150 °C, the reduction rate of Zn increases to 86.95%, which means that the increased reduction temperature can greatly enhance the reduction of zinc ash and the volatilization rate of zinc vapor. When the temperature rises to 1200 °C, reduction rate of Zn increases to 92.58%, only a small amount of zinc oxide powder covers the surface of the reduced pellets. When the reduction temperature increases up to 1250 °C, reduction rate of Zn increases to 97.75%, only minimal ZnO residues are observed, and a small amount of the liquid phase turning into sintered blocks is also observed, which demonstrates that the rapid reduction of carbon-containing zinc ash pellets contributes to the fast evaporation of the reduced zinc vapor, instead of oxidization on the surface of the pellets. Therefore, the optimal reduction temperature should be controlled below 1250 °C, to avoid the formation of a large amount of the liquid phase and to obtain a higher reduction rate.

### 3.3. Effect of Time on Reduction Rate of Zinc Oxide

**Figure 7:**
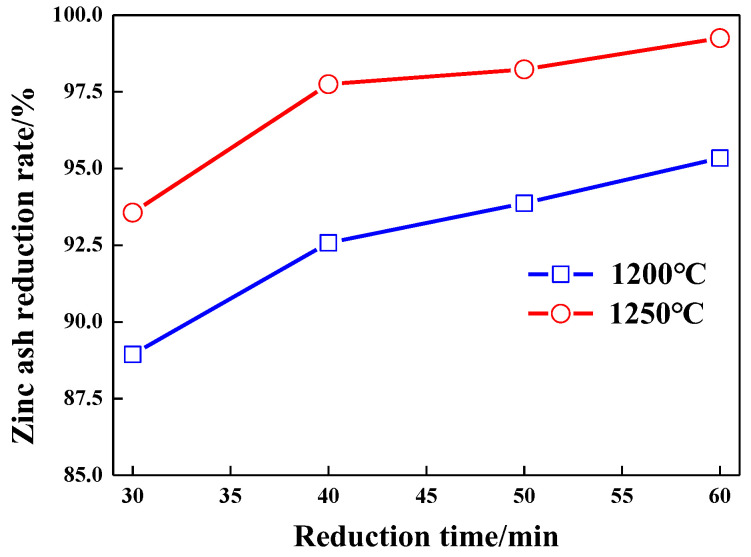
Effect of time on reduction rate of zinc ash.

Reduction time affects not only the reduction rate of Zn in carbon-containing pellets but also the smelting efficiency and energy consumption [12,15,23,24,25,26,27]. The relation of the ZnO reduction rate of zinc ash pellets and the different reduction times (30 min, 40 min, 50 min, and 60 min) are investigated, as shown in Figure 7. It can be seen that the reduction time exhibits great influence on the reduction rate of the zinc ash pellets, so the longer the reduction time is, the higher the Zn-reduction rate. When the reduction temperature is 1200 °C, the growth rate of the zinc-reduction rate is about 1.6%, with the increase in reduction time. When the reduction temperature is 1200 °C, the growth rate of the zinc-reduction rate is about 1.42%. When the reduction time is 30 min, reduction temperatures are 1200 °C and 1250 °C, and the reduction rates of Zn are 88.94% and 93.56%, respectively. When the reduction time increases to 50 min, the reduction rates of Zn increase up to 93.87% and 98.23%, respectively. When the reduction time increases to 50 min, the reduction rates of Zn increase up to 95.34% and 99.25%. Therefore, increasing the reduction temperature and time can significantly improve the reduction rate of zinc. Based on the test results from the different reduction times, the reduced pellets are covered with white ZnO powder after reduction at 1200 °C, which is caused by the oxidization of zinc vapor. However, no ZnO residue is found on the surface of reduced pellets after reduction at 1250 °C, which indicates that the higher reduction temperature increases the vapor pressure of zinc and accelerates the evaporation of zinc. On the basis of the Zn-reduction rate and industrial application feasibility, the optimal reduction temperature and time of zinc ash pellets are finally specified as 1250 °C and 60 min.

### 3.4. Effect of Reducing Agent Dosage on Reduction Rate of Zinc Oxide

**Figure 8:**
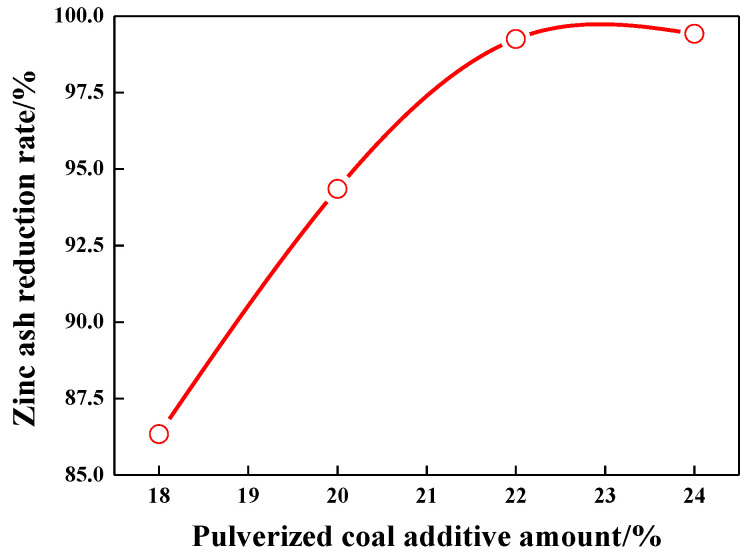
Effect of coal additive amount on reduction rate of zinc ash.

As a reducing agent, coal powder is used in carbon-containing zinc ash pellets. When the reduction temperature is 1200 °C, and the reduction time is 60 min, the effects of different coal content (18 wt.%, 20 wt.%, 22 wt.%, and 24 wt.%) on the reduction rate of zinc oxide were studied. Figure 8 shows the effect of coal additive amount on reduction rate of zinc ash. It can be seen that with the increase in pulverized coal content, the reduction rate of zinc oxide increases significantly, and excessive coal dosage wastes energy and consumes more heat. Therefore, it is necessary to find the premium coal content.

As presented in Figure 8, as the coal additive increases, Zn reduction in zinc ash rises gradually and becomes stable with a dosage of up to 22%. At 18% dosage, an 86.34% Zn reduction is tested. According to the calculation of a 1:1 mole ratio of oxygen in ZnO and FeO and fixed carbon in the coal powder, coal additive amounts to about 18%, theoretically, although it is up to the demand for coal addition in theory, as the loss of ignition (LOI) of carbon-containing pellets in high-temperature heating causes inadequate carbon addition and low Zn reduction. As the coal is added to 20 wt.%, Zn reduction grows to 94.35%, about 8% more, which means that the increased coal addition can greatly enhance Zn reduction. When the coal addition increases up to 22%–24 wt.%, the reduction rate of zinc oxide reached 99.25% to 99.42%, which indicates that when the amount of pulverized coal is 22 wt.%, the carbon amount in pellets is already enough to meet ZnO reduction in zinc ash, and more coal addition makes little difference to Zn-reduction enlargement. On the contrary, excessive pulverized coal will only increase coal consumption and reduce energy efficiency.

**Figure 9:**
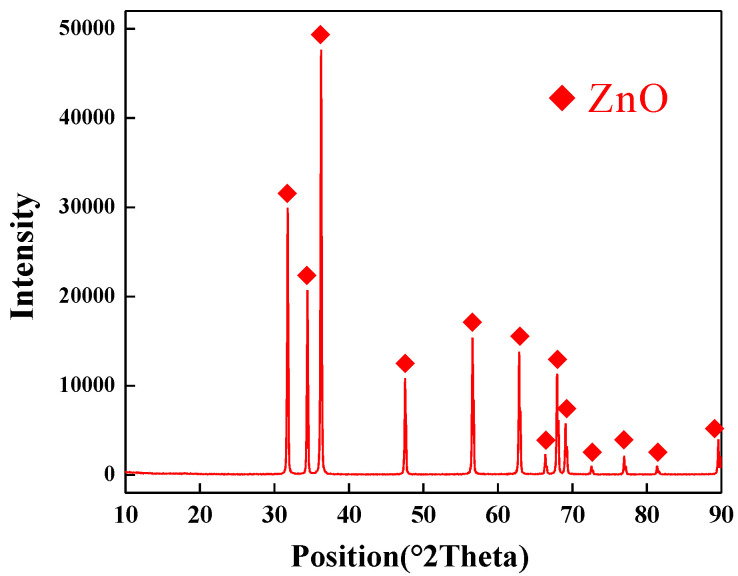
XRD pattern of ZnO product.

**Figure 10:**
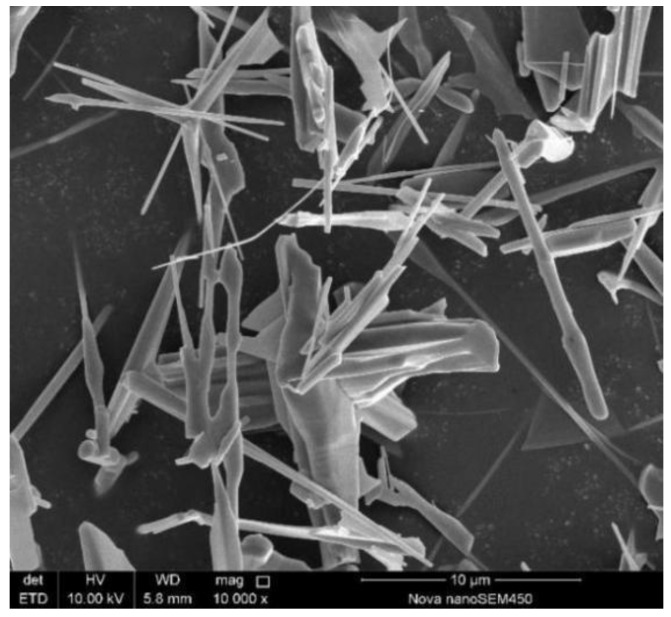
SEM images of ZnO product.

Under the optimal process conditions, a ZnO product with a purity of 99.5% is obtained. Figure 9 presents the XRD pattern of the ZnO product, and the SEM images of the ZnO product is shown in Figure 10. Due to the extremely high purity of the zinc oxide products, no other material phases are observed in the XRD pattern. SEM image shows that the zinc oxide product has a needle-like crystal structure with a length of 10 μm. Therefore, the high-purity zinc oxide obtained by direct reduction can replace the zinc oxide produced by the indirect process and can be used as a rubber vulcanizing agent, zinc oxide arrester, manganese zinc soft magnetic material, etc.

## 4. Conclusions

(1) Zn in zinc ash mainly exists in the form of zinc oxide (ZnO), basic zinc chloride (Zn_5_(OH)_8_Cl_2_H_2_O), and metallic zinc (Zn). The optimal conditions for zinc ash dechlorination are determined as a roasting temperature of 1000 °C and a roasting time of 60 min. After roasting, Cl content in zinc ash is only 0.021 wt.%, and the removal rate of the Cl element reached 99.5%.

(2) The reduction test of carbon-containing zinc ash pellets shows that the reduction temperature has the most influence on the reduction rate of zinc oxide. The optimal process parameters are a reduction temperature of 1250 °C, a reduction time of 60 min, and a coal powder content of 22 wt.%. Under the optimal process conditions, the reduction rate of Zn in zinc ash reaches 99.25%, and the ZnO content increases up to 99.5 wt.%. The ZnO content of the product is close to that of indirect zinc oxide.

(3) Due to the extremely high purity of the zinc oxide products, no other material phases are observed in the XRD pattern. SEM image shows that the zinc oxide product has a needle-like crystal structure with a length of 10 μm. The high-purity zinc oxide obtained by direct reduction can replace the zinc oxide produced by the indirect process and can be used as a rubber vulcanizing agent, zinc oxide arrester, manganese zinc soft magnetic material, etc.

## Figures and Tables

**Table 1:** Chemical composition of zinc ash, wt.%.

Component	ZnO	Cl	Fe_2_O_3_	SiO_2_	CaO	MgO	Al_2_O_3_
Content	87.91	4.44	0.26	1.27	0.49	0.23	0.50

**Table 2:** Chemical composition of powder coal, wt.%.

Component	C	Ash	Volatiles	S	P	H_2_O
Content	78.73	10.16	8.55	0.28	0.023	2.56

**Table 3:** Chemical analyses of zinc ash after dechlorination, wt.%.

Component	ZnO	Cl	Fe_2_O_3_	SiO_2_	CaO	MgO	Al_2_O_3_
Content	95.05	0.021	0.35	1.68	0.63	0.42	0.73

## Data Availability

Not applicable.

## References

[1] Zhang Y.Y., Yu L.H., Fu T., Wang J., Shen F.Q., Cui K.K. (2022). Microstructure evolution and growth mechanism of Si-MoSi_2_ composite coatings on TZM (Mo-0.5Ti-0.1Zr-0.02 C) alloy. J. Alloys Compd..

[2] Cui K.K., Mao H.B., Zhang Y.Y., Wang J., Wang H., Tan T.B., Fu T. (2022). Microstructure, mechanical properties, and reinforcement mechanism of carbide toughened ZrC-based ultra-high temperature ceramics: A review. Compos. Interfaces.

[3] Zhang Y.Y., Fu T., Yu L.H., Shen F.Q., Wang J., Cui K.K. (2022). Improving oxidation resistance of TZM alloy by deposited Si-MoSi_2_ composite coating with high silicon concentration. Ceram. Int..

[4] Zhang Y.Y., Yu L.H., Fu T., Wang J., Shen F.Q., Cui K.K., Wang H. (2022). Microstructure and oxidation resistance of Si-MoSi_2_ ceramic coating on TZM (Mo-0.5Ti-0.1Zr-0.02C) alloy at 1500 °C. Surf. Coat. Technol..

[5] Fu T., Shen F., Zhang Y., Yu L., Cui K., Wang J., Zhang X. (2022). Oxidation protection of high-temperature coatings on the surface of Mo-based alloys—A Review. Coatings.

[6] Mao H.B., Shen F.Q., Zhang Y.Y., Wang J., Cui K.K., Wang H., Lv T., Fu T., Tan T.B. (2021). Microstructure and mechanical properties of carbide reinforced TiC-based ultra-high temperature ceramics: A Review. Coatings.

[7] Doustkhah E., Esmat M., Fukata N., Ide Y., Hanaor A.H., Assadi M.N. (2022). MOF-derived nanocrystalline ZnO with controlled orientation and photocatalytic activity. Chemosphere.

[8] Shafi M.A., Bouich A., Fradi K., Guaita J.M., Khan L., Mari B. (2022). Effect of deposition cycles on the properties of ZnO thin films deposited by spin coating method for CZTS-based solar cells. Optik.

[9] Guaita J.M., Bouich A., Mari B. (2021). Shedding light on phase stability and surface engineering of formamidinium lead iodide (FaPbI_3_) thin films for solar cells. Eng. Proc..

[10] Wang J., Zhang Y.Y., Yu L.H., Cui K.K., Fu T., Mao H.B. (2022). Effective separation and recovery of valuable metals from waste Ni-based batteries: A comprehensive review. Chem. Eng. J..

[11] Sharma P., Hasan M.R., Mehto N.K., Deepak, Bishoyi A., Narang J. (2022). 92 years of zinc oxide: Has been studied by the scientific community since the 1930s-An overview. Sens. Int..

[12] Kołodziejczak-Radzimska A., Jesionowski T. (2014). Zinc oxide—From synthesis to application: A review. Materials.

[13] Hirase R., Nagatani A., Yuguchi Y. (2020). Development of powdering method for cellulose nanofibers assisted by zinc oxide for compounding reinforced natural rubber composite. Curr. Res. Green Sustain. Chem..

[14] Ahmed N.M., Nashar D.E.E.I. (2013). The effect of zinc oxide-phosphate core-shell pigments on the properties of blend rubber composites. Mater. Des..

[15] Moezzi A., McDonagh A.M., Cortie M.B. (2012). Zinc oxide particles: Synthesis, properties and applications. Chem. Eng. J..

[16] Zuo X., Yoon S.D., Yang A. (2009). Ferromagnetism in pure wurtzite zinc oxide. J. Appl. Phys..

[17] Wang J., Zhang Y.Y., Cui K.K., Gao T.F.J.J., Hussain S., AlGarni T.S. (2021). Pyrometallurgical recovery of zinc and valuable metals from electric arc furnace dust-A review. J. Clean. Prod..

[18] Anandaraj S., Karthik S., Vijaymohan S., Rampradheep G.S., Indhiradevi P., Anusha G. (2022). Effects of using white flour, zinc oxide and zinc ash as an admixture in mortar and concrete. Mater. Today: Proc..

[19] Garg R., Garg R. (2021). Effect of zinc oxide nanoparticles on mechanical properties of silica fume-based cement composites. Mater. Today: Proc..

[20] Kumar M., Bansal M., Garg R. (2021). An overview of beneficiary aspects of zinc oxide nanoparticles on performance of cement composites. Mater. Today: Proc..

[21] Kang Y., Yu F., Zhang L., Wang W.H., Chen L., Li Y.C. (2021). Review of ZnO-based nanomaterials in gas sensors. Solid State Ion..

[22] Shamsipur M.A., Pourmortazavi S.M., Hajimirsadeghi S.S. (2013). Facile synthesis of zinc carbonate and zinc oxide nanoparticles via direct carbonation and thermal decomposition. Ceram. Int..

[23] Zhu D.Q., Wang D.Z., Pan J. (2021). A study on the zinc removal kinetics and mechanism of zinc-bearing dust pellets in direct reduction. Powder Technol..

[24] Wang H.Q., Li C.H., Zhao H.G. (2013). Preparation of nano-sized flower-like ZnO bunches by a direct precipitation method. Adv. Powder Technol..

[25] Rudnik E. (2020). Hydrometallurgical recovery of zinc from industrial hot dipping top ash. Trans. Nonferrous Met. Soc. China.

[26] Rudnik E. (2019). Recovery of zinc from zinc ash by leaching in sulphuric acid and electrowinning. Hydrometallurgy.

[27] Wang S.X., Xu C.Y., Lei Z., Li J.R., Lu J.L., Xiang Q.Q., Chen X., Hua Y.X., Li Y. (2022). Recycling of zinc oxide dust using ChCl-urea deep eutectic solvent with nitrilotriacetic acid as complexing agents. Miner. Eng..

